# Dynamic Impacts of Stock Enhancement on Kaluga Sturgeon (*Huso dauricus*): Novel Conservation Strategy Insights from the Gut Microbe Composition and Gene Expression Mode

**DOI:** 10.3390/ijms26041480

**Published:** 2025-02-10

**Authors:** Yutao Li, Ruoyu Wang, Cunhua Zhai, Dingchen Cao, Zhipeng Sun, Ying Zhang, Bo Ma

**Affiliations:** 1 Key Laboratory of Cold Water Fish Germplasm Resources and Multiplication and Cultivation of Heilongjiang Province, Heilongjiang River Fishery Research Institute, Chinese Academy of Fishery Sciences, Harbin 150070, China; liyutao@neau.edu.cn (Y.L.); wangruoyu@hrfri.ac.cn (R.W.); caodingchen@hrfri.ac.cn (D.C.); 2 Heilongjiang River Basin Fishery Resources and Environment Scientific Observation Experimental Station, Heilongjiang River Fishery Research Institute, Chinese Academy of Fishery Sciences, Harbin 150070, China; cunhuazhai@hrfri.ac.cn (C.Z.); sunzhipeng@hrfri.ac.cn (Z.S.)

**Keywords:** stock enhancement, sturgeon, environmental adaptation, gut microbiota, conservation biology

## Abstract

The sturgeon population has experienced strict threats due to inordinate human activities in the last decade and has been classified into the Red List of Threatened Species in recent years. Stock enhancement is one effective practice for the conservation of wild sturgeons. However, the survival conditions for sturgeon were not satisfactory after they were directly restocked into their natural habitat. *Huso dauricus* is an important protected sturgeon species, and finding an appropriate conservation strategy for the wild population is urgent. To clarify the dynamic adaptability of *Huso dauricus* to its wild environment, 1000 individuals were released into a natural river. On the 0th, 7th, 14th, and 30th days, five recaptured individuals were used to evaluate the dynamic trends in biochemical biomarkers, intestinal histomorphology, gut microbe taxon composition, and transcription profile over 30 days of stock enhancement. Our results indicated that *Huso dauricus* individuals still had a physiological stress status on the seventh day and then gradually adapted to the wild habitat 14 days after reintroduction based on the serum cortisol level. Meanwhile, the feeding habitat, organ function indicators, and growth performance showed similar dynamic changes within 30 days. Interestingly, their gut bacterial diversity and taxon structure also fluctuated over the 30 days after restocking, and they were accompanied by dynamic changes in intestinal pathological injury and tight junction protein expression in this period. The transcriptome analysis revealed the dynamic adaptability of *Huso dauricus* to wild habitats associated with the expression modes of genes related to the FoxO family, immune system, cytochrome family, and ATP metabolism. Taken together, the findings of the present research demonstrated that artificial reintroduction had dynamic impacts on the health condition of *Huso dauricus* and that 14 days of wilderness training might be essential for sturgeon restocking practices. Our study revealed the adaption mechanism of *Huso dauricus* at the molecular level during the restocking period and shed light on the theoretical guidelines for wild sturgeon conservation.

## 1. Introduction

Sturgeon represents the general term for subcold-water Acipenseriformes taxa and can be divided into two main taxonomical classifications based on geologic age. One comprises the extinct ancient sturgeon taxon, and the other includes the latter-day sturgeon population that originated in the Cretaceous period 200 million years ago. Latter-day sturgeon species include two families (Acipenseridae and Polyodontidae), six genera [1], and 27 species [2] and have been regarded as living fossils [3]. The *Huso* and *Acipenser* genera are two representative taxa in the existing sturgeon population. The sturgeon population is the oldest potamodromous bony fish distributed in lakes, rivers, and oceans of the Northern Hemisphere, including Eastern Europe, East Asia, and the east coast of North America [4]. In China, eight sturgeon species are mainly distributed in the Yangtze River (e.g., *Acipenser sinensis*), Amur River (e.g., *Huso dauricus*), and Ili/Ertix River in Xinjiang (*Acipenser nudiventris* and *Acipenser ruthenus*). Un-fortunately, wild sturgeon populations have constantly declined in the last decade due to drastic caviar market demand, dam and hydro-facility construction, environmental emissions of artificial contaminants [5], and global climate change [6]. Meanwhile, expanding the scale of the sturgeon population in the short term is difficult due to the lengthy sexual maturity cycle of female individuals [7]. Therefore, wild sturgeon was listed in the Convention on International Trade in Endangered Species in 1998 [8] and subsequently divided into critically endangered protected species. Local authorities in China corroborated that *Psephurus gladius* became extinct in 2022 [2]. It is apparent that an efficient conservation strategy is urgent for the restoration of the wild sturgeon population. Germany executed a remediation plan to sustain the population scale of *Acipenser oxyrinchus* in the Baltic by restocking its natural habitats beginning in 1996 [9]. In China, profitable sturgeon fishing activity was strictly prohibited in the Yangtze River, and the government decided to reconstruct a stable wild population of *Acipenser dabryanus* within 15 years [10].

To safeguard wild sturgeons against the negative stress derived from their natural habitats, people usually breed this endangered species in an artificial environment. A series of monitoring investigations also revealed that the captive breeding model could provide beneficial living conditions for endangered wildlife [11] and attenuate extinction risk [12]. Nevertheless, artificial breeding practices force wildlife to adapt to living conditions (such as diet structure and living space) that are greatly different from those in their natural environment [13]. Thus, long-term hatchery breeding might induce serious threats to the health of wild sturgeon because prolonged stimulus deficiency would affect the plasticity of the nervous system, in turn reducing the fitness and survival capacity of the species [14,15]. Previous research found that artificial aquaculture practices visibly decreased the food exploration efficiency of *Acipenser oxyrinchus* [6] and caused a natural decline in fertility [16]. Artificial stock enhancement in rivers could be regarded as a communication bridge between the wild sturgeon population and the artificially bred population [17]. The reintroduction of endangered sturgeons to their natural habitat not only directly supplements population scales but also maintains the homeostasis and biodiversity/genetic diversity of freshwater ecosystems and ensures the sustainable development and utilization of aquatic bio-resources [18]. In sturgeon conservation biology, it is even thought that population stock enhancement has greater ecological value than single fishing bans [8]. Obviously, artificial stock enhancement is an essential method for restoring the wild sturgeon population. A series of restocking activities for sturgeon have been implemented effectively since 1955, and more than 90 million sturgeon per year have been released into the Caspian Sea and Missouri River [19]. However, detailed restocking periods for sturgeon still need to be explored because inopportune rewilding might increase aquaculture inputs and affect the survival capacity of sturgeon species [20]. Some studies in the literature found that only 3% of sturgeon individuals could survive in a wild environment without wilderness training during the stock enhancement period [10,21].

The intestinal micro-organism community is composed of various taxa and is mixed in proportions to form an important source of genetic/metabolic diversity in fish [22]. Gut bacteria can be regarded as the most abundant microbe taxa in the intestinal micro-environment, with more than 10^14^ species [23], and they modulate innate immune activation [1], energy storage, nutrition absorption, and glycometabolism [24,25]. Moreover, enteric colonization with beneficial bacteria would allow competition with harmful pathogens for limited niche space and nutrients to enhance the ecological adaption of the host [26,27]. The conversion of external environmental factors (such as food sources and habitat locations) could evoke a succession of gut microbe communities on a temporary time scale [28]. It can be seen that the re-wilding process might affect the gut bacterial composition and physiological functions because of habitat conditions and diets that differ from those in the artificial rearing environment [29]. High-throughput sequencing was used to characterize the differences in intestinal microbe taxa between domestic and wild *Centropomus undecimalis* and *Acipenser ruthenus* [1]. The gut bacterial community usually dynamically changes together with the extension of the artificial reintroduction process [30]. Accordingly, an appropriate stock enhancement project for sturgeon should be implemented when the individuals used for rewilding possess differential microbial compositions from those in the artificial breeding environment and maintain a relatively stable community structure.

The process of an organism’s adaptation to an external environment usually depends on changes in the transcription levels of specific genes [31]. However, few studies have utilized transcriptomics sequencing to reveal the differences in the gene expression profiles of sturgeon during the artificial stock enhancement period. *Huso dauricus* is a critically endangered sturgeon species with important scientific research value and a massive body size [32]. In China, wild *Huso dauricus* individuals were mainly found in the drainage basin of the Songhua River and Wusuli River, which runs through Russia and Heilongjiang Province (China). Due to inordinate human activities, wild *Huso dauricus* individuals are still threatened in their natural habitat, and the population scale has declined in the last decade. Therefore, it is urgent to elevate the effectiveness of stock enhancement to expand the wild population scale of *Huso dauricus*. Our study integrated microbiomics, transcriptomics, histomorphology, and biochemical analysis to implement a comprehensive evaluation of the dynamic impacts of artificial reintroduction on *Huso dauricus*. The present investigation not only provides a suitable conservation strategy for *Huso dauricus* but also paves the way for further research on the adaption mechanism of wild sturgeon during the restocking period.

## 2. Results

### 2.1. Effects of Stock Enhancement on the Growth Performance of Kaluga Sturgeon

The D7FL, D14FL, and D30FL groups displayed no obvious changes in the ratio of body weight and body length compared with the Ori group (*p* > 0.05), indicating that stock enhancement did not markedly affect their growth and development (Table S1). In addition, it was observed that body weight decreased 7 d after restocking in the river habitat and then rose steadily after 14 d of stock enhancement.

### 2.2. Effects of Stock Enhancement on the Feeding Habits of Kaluga Sturgeon

Table S1 shows that the gut content weight in the D7FL group was significantly lower than that in the Ori group (*p* < 0.05), suggesting that Kaluga sturgeon could not obtain adequate food resources from the natural environment in the initial stock enhancement stage. The gastric contents in the D7FL group were consistent with the above result, with fewer prey species (Table S2).

It was found that there was obvious feed residue in the stomach at 0th d. The Kaluga sturgeon had captured some small-sized fish from natural river areas, but most individuals could not forage successfully. As expected, the food composition became more abundant from 14 d to 30 d (Table S2). Some mussel and pond crayfish debris could be identified in the gastric cavity after 14/30 d of re-wilding; in parallel, river snails were also found in residual stomach contents that were collected on the 30th d.

### 2.3. Effects of Stock Enhancement on the Biochemical Biomarkers of Kaluga Sturgeon

The blood cortisol level can be regarded as a critical biomarker for stress status assessment in mammals. Compared with the Ori group, in the D14FL and D30FL groups, the cortisol content in serum was significantly elevated at 7 d after restocking (*p* < 0.05). Meanwhile, there was no statistical difference between the Ori group and the D14FL/D30FL group (*p* > 0.05), indicating that a physiological stress response was induced in the initial re-wilding period (within 7 d) and was then gradually restored to the normal condition from the 14th d (Table S1).

Although the above results indicated that foraging conditions were different in different stock enhancement periods, the blood glucose level was not statistically significantly different among the four groups (Table S1). This could imply that the dynamic food intake levels over the 30 d of re-wilding did not induce carbohydrate metabolism disorder.

Electrolyte homeostasis represents a normal ion distribution in the intracellular/extracellular environment, and this reflects the comprehensive metabolic status of various organs. Our study illustrated that restocking in a wild habitat might have significantly decreased the blood potassium content within 30 d compared with that in the Ori group (*p* < 0.05), but it might have been gradually restored to the normal level with an increasing trend (Table S1). In addition, the blood phosphorus concentration in D7FL/D14FL groups was obviously lower than that in the Ori group (*p* < 0.05) and began to recover to the normal level on the 30th d of the re-wilding period.

Excessive fat accumulation in blood can injure normal organ functions. The serum cholesterol concentration in the D14FL group was markedly higher than that in the Ori group (*p* < 0.05); then, it was almost reduced to the initial level on the 30th d. Compared with the initial conditions before artificial stock enhancement (0th d), restocking in the natural river habitat significantly elevated the high-density lipoprotein cholesterol content (*p* < 0.05) with no obvious time-dependent effects.

Changes in survival environment conditions might generate a series of negative impacts on organ functions (Table S1). Compared with the D14FL group, the carbon dioxide combining power in the other three groups displayed an obvious decreasing trend (*p* < 0.05), indicating that the kidneys released less bicarbonate radical to induce compensatory alkali accumulation during the stock enhancement period. Meanwhile, creatine kinase in D30FL was higher than that in the Ori and D7FL groups (*p* < 0.05), implying that *Huso dauricus* might require more adenosine triphosphate (ATP), which is produced by the myocardium after sufficient adaption to habitats in natural water areas. Liver function indicators can be regarded as the assessment standard for the hepatic metabolism capacity. Compared with the Ori group, alanine aminotransferase in serum was elevated significantly after artificial stock enhancement in the wild habitat (*p* < 0.05). Similarly, aspartate aminotransferase showed an increasing trend in the D30FL group compared with the Ori and D7FL groups (*p* < 0.05). Interestingly, the alkaline phosphatase level in serum rose continuously from the 7th d to the 30th d, and an obviously significant difference was displayed between the Ori and D14FL/D30FL groups. It was also observed that the serum total protein and lactate dehydrogenase significantly changed on the 7th d and 14th d, respectively, compared with those in the D30FL group (*p* < 0.05). In parallel, there was no statistically significant difference between the D30FL group and the Ori group (*p* > 0.05), which indicated that short-term re-wilding affected normal liver functions to a certain extent, while the 30 d restocking process alleviated the physiological stress response caused by abrupt, artificial stock enhancement.

### 2.4. Effects of Stock Enhancement on the Enteric Tight-Junction Protein Expression in Kaluga Sturgeon

**Figure 1:**
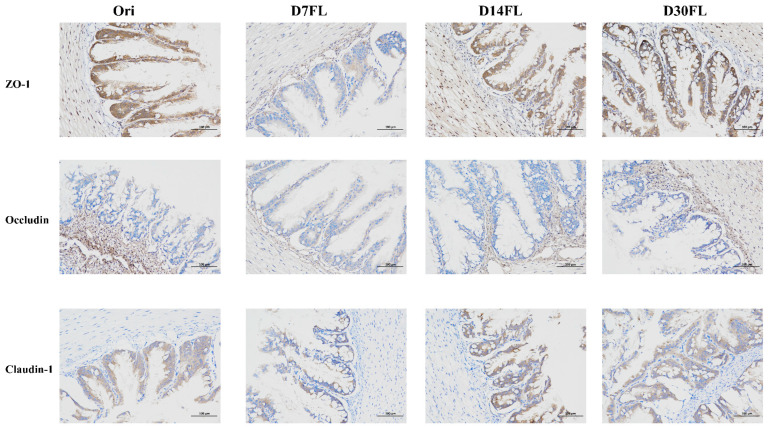
Positive expression area of intestinal tight-junction proteins (ZO-1, Occludin, and Claudin-1) in *Huso dauricus*. The brown area represents the target proteins, while the blue area represents the cell nuclei.

An immunohistochemical technique was utilized to evaluate the intestinal barrier function based on the expression levels of tight-junction proteins (Occludin, Claudin-1, and ZO-1) (Figure 1). Compared with those in the Ori group, the translation levels of the three typical tight-junction proteins on the mucosal surface were obviously inhibited on the 7th d. Interestingly, the positive expression levels of these tight-junction proteins began to display an increasing trend from the 14th d. After 30 d of restocking adaption, the gut barrier biomarker levels tended to recover to a nearly normal status.

### 2.5. Effects of Stock Enhancement on the Gut Bacterial Taxa in Kaluga Sturgeon

**Figure 2:**
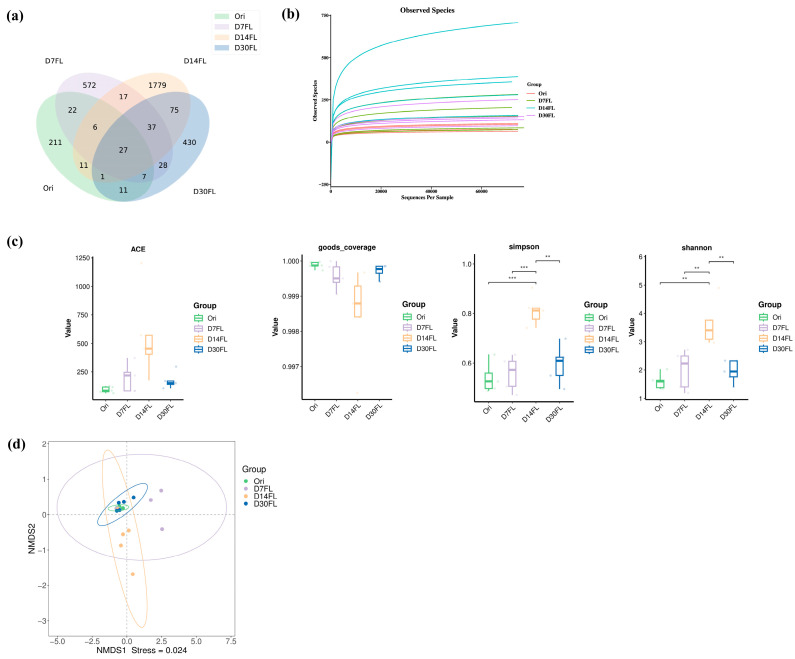
Overview of the ASV feature sequence statistics. (**a**) Venn diagram of the ASV feature sequence numbers. Green: Ori group. Purple: D7FL group. Orange: D14FL group. Blue: D30FL group. (**b**) Rarefaction plot of the observed species numbers. Red: Ori group. Green: D7FL group. Blue: D14FL group. Purple: D30FL group. (**c**) α-diversity indexes (ACE, goods_coverage, Simpson, and Shannon). Green: Ori group. Purple: D7FL group. Orange: D14FL group. Blue: D30FL group. Pairwise comparisons were performed using a *t*-test to compare the significance levels among the four groups (*n* = 5). **: *p* < 0.01; ***: *p* < 0.001. (**d**) NMDS analysis based on the weighted Unifrac distance algorithm (*n* = 5). Green: Ori group. Purple: D7FL group. Orange: D14FL group. Blue: D30FL group.

After the raw sequencing data were filtered, denoised, and merged and chimera sequences were deleted, we found that the validity ratio for all samples ranged from 93.49 to 97.15% (Table S3). Therefore, our sequencing data used for the subsequent bioinformatic analysis had eligible credibility. In addition, a total of 4399 ASVs were identified from 20 individuals. Among them, there were 211, 572, 1779, and 430 unique ASV features in the Ori, D7FL, D14FL, and D30FL groups, while 27 shared ASV sequences were found between the four groups (Figure 2a). Based on the rarefaction plot (Figure 2b), each curve displayed a nearly flat trend, which suggested that the current sequencing project had sufficient depth to identify all existing species. For the α-diversity indexes (Figure 2c), it was found that the ACE and goods_coverage indexes did not have obvious differences among the four groups. Conversely, the Shannon and Simpson indexes showed an extremely distinct significance between the D14FL group and the other three groups (*p* < 0.01). As an important signature of differences in the structure of the microbiota, β-diversity parameters such as NMDS analysis showed an obvious tendency to separate in the four groups (stress coefficient < 0.05, Figure 2d). Our results showed that the bacterial taxa had a greater distance between the Ori and D7FL groups. In parallel, the D30FL group had a closer matrix distance to that of the Ori group, indicating that the overall microbial composition was nearly restored to the original status 30 d after restocking.

**Figure 3:**
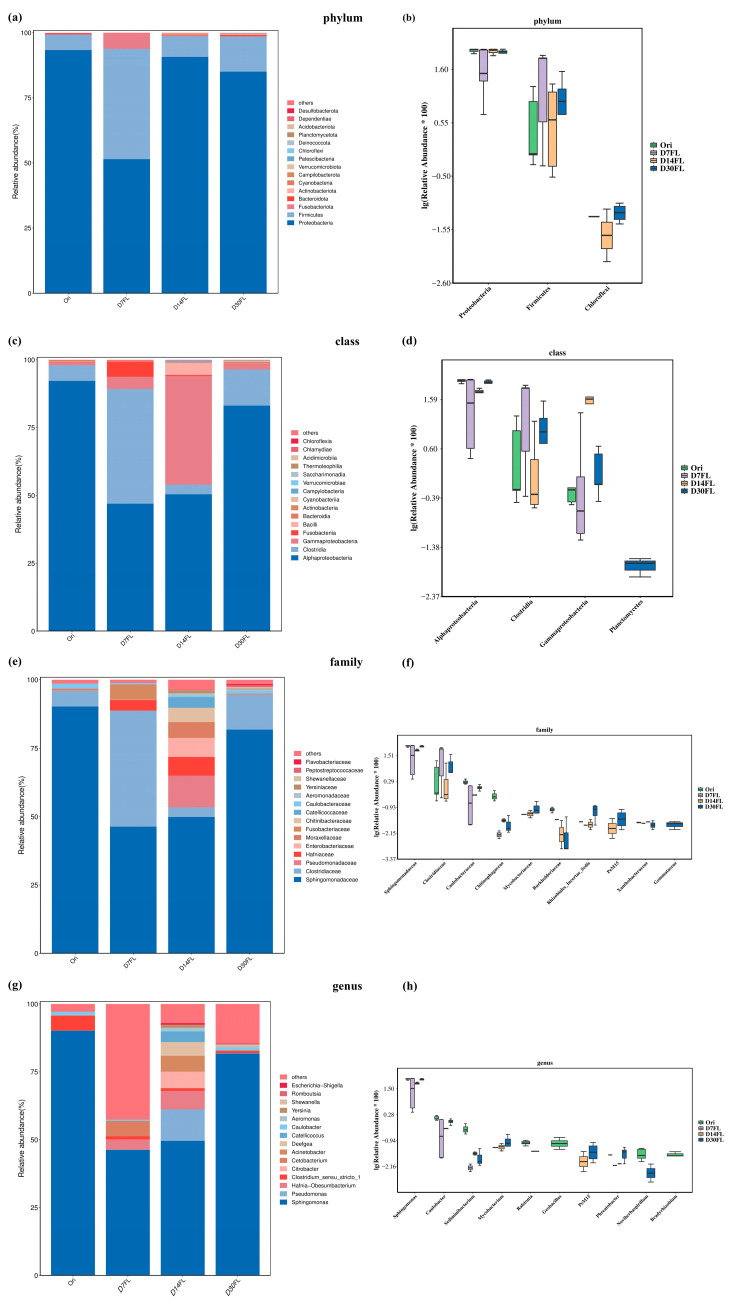
Taxonomical classification of the intestinal bacterial community in Kaluga sturgeon. A one-way analysis of variance with Tukey’s multiple comparisons was utilized to evaluate the significant differences among the four groups (*n* = 5). (**a**) Stacked bar chart for the phylum level. (**b**) Boxplot of the differential taxa based on the phylum level. (**c**) Stacked bar chart for the class level. (**d**) Boxplot of the differential taxa based on the class level. (**e**) Stacked bar chart for the family level. (**f**) Boxplot of the differential taxa based on the family level. (**g**) Stacked bar chart for the genus level. (**h**) Boxplot of the differential taxa based on the genus level.

Detailed species number statistics for different taxonomic levels in 20 samples can be seen in Figure S1. For the phylum level, more than 93% of the bacterial taxa could be classified into the Proteobacteria and Firmicutes phyla (Figure 3a). Interestingly, the relative abundance of Fusobacteriota in the D7FL group was obviously higher than that in the other three groups, but a significant difference among these four groups was not observed (*p* > 0.05, *p* value = 0.43). Among the top 15 phyla of the microbiota, Proteobacteria, Chloroflexi, and Firmicutes were found to be statistically significant among the four groups (*p* < 0.05). Seven days after stock enhancement, the abundance of Proteobacteria decreased significantly, while Firmicutes taxa were more inclined to colonize the intestinal canal of Kaluga sturgeon than in the Ori, D14FL, and D30FL groups (Figure 3b).

For the class level, the relative abundance of Alphaproteobacteria was significantly decreased after 7 d of re-wilding (*p* < 0.05) and was then gradually restored to a nearly normal level from 14 to 30 d (Figure 3d). On the contrary, the mean relative abundance of the Gammaproteobacteria class (an important member of the Proteobacteria phylum) continuously increased from the 0th to the 14th d of the stock enhancement process (*p* < 0.01). Subsequently, it was nearly restored to the initial level (0th d) on the 30th d (Figure 3d). Similarly, the Clostridia class also displayed complex change dynamics during the 30 d stock enhancement period. Seven days after restocking, its relative abundance increased to nearly 7.5-fold (D7FL), then declined to the initial level (D14FL), and finally increased significantly compared with the Ori group (D30FL, *p* < 0.05). Coincidentally, the Gammaproteobacteria, Alphaproteobacteria, and Clostridia classes were also dominant taxa in the gut bacterial community of Kaluga sturgeon (Figure 3c). Interestingly, an important type of aquatic microbiota, the Planctomycetes class, could only be identified in the intestinal micro-environment after 30 d of stock enhancement, implying that Kaluga sturgeon could build stable microbial communication with the external environment after 1 month.

Although 10 families were identified as the differential microbiota community based on mathematical statistics (*p* < 0.05), only the Sphingomonadaceae and Clostridiaceae families had representative abundance values in the four groups (Figure 3e,f). Therefore, it might be inferred that these two families were sensitive to the changes in the external environment and were involved in the establishment of adaptation to the natural habitat during stock enhancement. Compared with the Ori group, 7 d of re-wilding inhibited the colonization conditions of Sphingomonadaceae taxa and markedly elevated the relative richness of Clostridiaceae (*p* < 0.05). Similarly, these two taxa were restored to the initial levels on the 14th and 30th d of the stock enhancement period, respectively.

Based on the taxonomical genus level, *Sphingomonas* was regarded as the critically predominant taxon in the intestinal microbe community of *Huso dauricus* and showed the same change trend as the Sphingomonadaceae family (Figure 3g,h). In addition, the relative abundance of the *Caulobacter* genus also had statistically significant differences among the four groups (*p* < 0.01) and constantly declined from the 0th to the 14th d after restocking. Subsequently, it increased to the initial level on the 30th d (Figure 3h).

**Figure 4:**
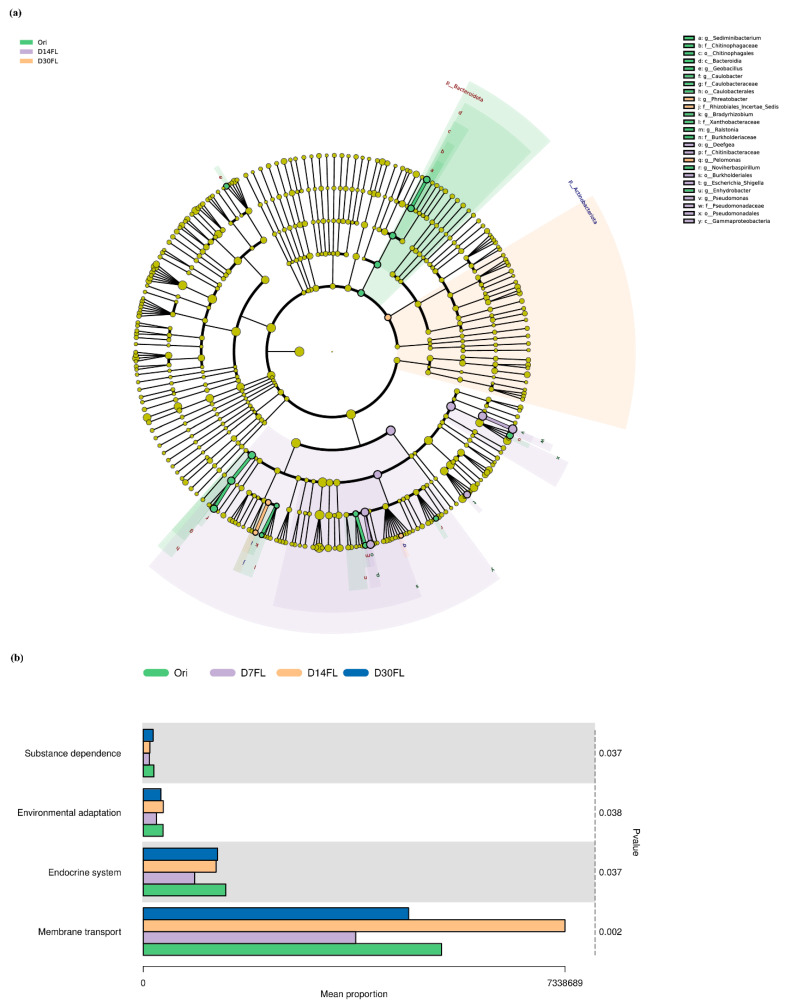
Functional analysis for differential microbiota. (**a**) Cladogram for LEfSe analysis. Green: Ori group. Purple: D14FL group. Orange: D30FL group. (**b**) KEGG pathway enrichment analysis. A Kruskal–Wallis test was applied to compare the significant differences among these four groups. Green: Ori group. Purple: D7FL group. Orange: D14FL group. Blue: D30FL group.

LEfSe analysis was utilized to filter the biomarkers in each group (Figure 4a). It was shown that the *Sediminibacterium*, *Geobacillus*, *Bradyrhizobium*, *Ralstonia*, *Noviherbaspirillum*, *Enhydrobacter,* and *Caulobacter* genera in the Ori group had typically dominant abundance, while the Pseudomonadales order, Pseudomonadaceae family, and *Pseudomonas* genus were regarded as representative biomarkers for the 14th d of the stock enhancement period. Meanwhile, the Rhizobiales_Incertae_Sedis family, *Phreatobacter* genus, and *Pelomonas* genus were relatively high-abundance taxa in the D30FL group.

KEGG enrichment analysis was used to predict the changes in the metabolic pathways of gut microbes. A total of four signaling pathways with significant differences were identified among the four groups (*p* < 0.05). Similarly, the relative proportions of these four pathways declined on the 7th d after restocking compared with those in the Ori group. Subsequently, the mean proportions of “Endocrine system”, “Environmental adaptation”, and “Membrane transport” began to rise to a normal level on the 14th d. Our results implied that 14 d of re-wilding could alleviate the stress status induced by artificial stock enhancement. However, the mean proportion of the “Substance dependence” pathway needed a 30 d adaption period to be restored to the original level (Figure 4b).

### 2.6. Effects of Stock Enhancement on the Enteric Transcriptome Overview in Kaluga Sturgeon

In total, there were 6123, 3533, and 42 differentially expressed genes in Ori vs. D14FL, Ori vs. D30FL, and D14FL vs. D30FL, respectively (Figure S2). This suggested that Kaluga sturgeon displayed gene expression homeostasis from the 14th to the 30th d of the re-wilding period, and the artificial stock enhancement process obviously altered the previous transcription profile mode in the intestine to adapt to wild survival conditions.

**Figure 5:**
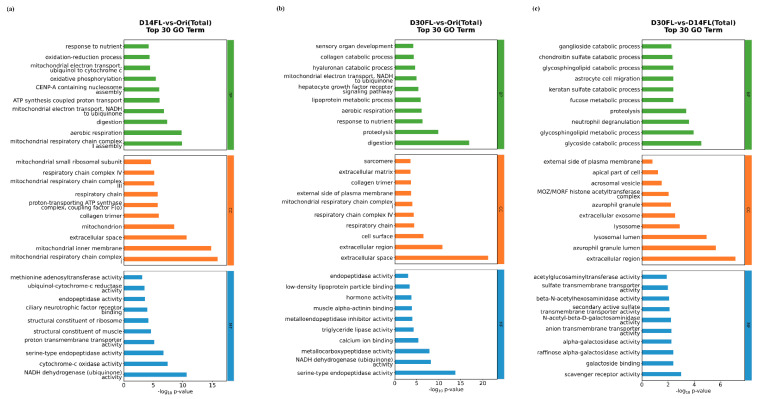
GO enrichment analysis for differentially expressed genes. The abscissa represents the -log_10_ (*p* value), while the ordinate represents detailed GO terms. Green: biological processes. Orange: cellular components. Blue: molecular functions. (**a**) Top 30 GO terms in D14FL vs. the Ori group. (**b**) Top 30 GO terms in D30FL vs. the Ori group. (**c**) Top 30 GO terms in D30FL vs. the D14FL group.

Differentially expressed genes between the Ori and D14FL groups could be mapped to 365 GO terms with statistical significance (*p* < 0.05). Among the top 30 GO terms with the most obvious significance, some GO terms related to nutrient absorption (such as “response to nutrient” and “digestion”) were enriched in biological processes (Figure 5a). In parallel, a series of GO terms related to mitochondrial energy production (such as “aerobic respiration”, “mitochondrial respiratory chain complex I”, and “NADH dehydrogenase (ubiquinone) activity”) dominated among cellular components, biological processes, and molecular functions (Figure 5a).

All differentially expressed genes that were identified between the Ori and D30 FL groups were annotated with 405 significant differences. Among them, 2263, 458, and 742 GO terms were associated with biological processes, cellular components, and molecular functions, respectively. Interestingly, “digestion” was also the dominant term for the biological processes, and typical GO terms related to cellular respiration (mitochondrial respiratory chain complex I, respiratory chain complex IV, and respiratory chain) were also enriched among the cellular components (Figure 5b). Meanwhile, “hormone activity” was a critical GO term that was classified among the top 10 most significant molecular function terms. Apart from the top 30 GO terms, various GO terms associated with immune response were also enriched between the Ori and D30FL groups (Table S4).

A total of 66 GO terms with significant differences were enriched between the D14FL and D30FL groups (Figure 5c). Compared with the other two groups, there were only a few GO terms related to exogenous stress response. For example, it was only found that GO terms related to lysosome phagocytosis (“lysosomal lumen” and “lysosome”) were enriched among the cellular components (Figure 5c).

**Figure 6:**
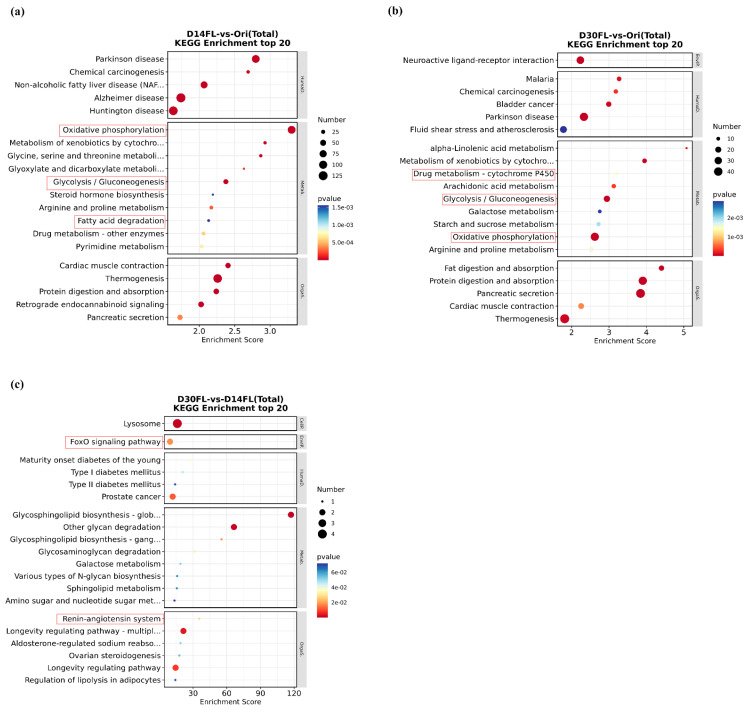
KEGG enrichment analysis of differentially expressed genes. The abscissa represents the enrichment score, while the ordinate represents the detailed KEGG pathways. The dot size represents the number of differentially expressed genes that were mapped to the specific KEGG pathways. The dot color, from blue to red, represents the enhancement of the significance level. KEGG pathways in the red box indicate the detailed signaling pathways associated with the stress-resistance process. (**a**) The top 20 KEGG pathways in D14FL vs. the Ori group. (**b**) Top 20 KEGG pathways in D30FL vs. the Ori group. (**c**) Top 20 KEGG pathways in D30FL vs. the D14FL group. KEGG pathways in the red frame represent the stress-resistance-related signaling pathways.

All differentially expressed genes (D14FL vs. Ori) were enriched in 44 significant KEGG pathways. Among them, typical pathways such as “Glycolysis/Gluconeogenesis”, “Fatty acid degradation”, and “Oxidative phosphorylation” were identified (Figure 6a). Interestingly, “Glycolysis/Gluconeogenesis” and “Oxidative phosphorylation” were also enriched between the Ori and D30FL groups (Figure 6b). Meanwhile, “Drug metabolism-cytochrome P450” was markedly enriched (*p* < 0.05). For the top 20 KEGG pathways that were enriched between the D14FL and D30FL groups, two significant signaling pathways (FoxO signaling pathway and renin-angiotensin system) that are involved in the self-adaption process to environmental stress were found (Figure 6c).

To explore the detailed regulatory mechanism of artificial stock enhancement in the physiological response in *Huso dauricus*, we selected nine typical genes involved in adenosine triphosphate (ATP) synthesis, the cytochrome (CYP) 450 family, and inflammation response based on the shared differentially expressed genes in the transcription profile (Figure S3). Compared with the Ori group, the mRNA levels of ATP5G, ATP6H, ATP15, CYP1A1, CYP1B1, and CYP27C1 displayed obvious trends of downregulation after 14–30 d of stock enhancement, while the interleukin (IL)-1β and MMP9 expression levels were elevated with the prolongation of the restocking period (14th–30th d). Interestingly, the expression trend of the ATP6 gene increased sharply first on the 14th d after stock enhancement and then significantly declined on the 30th d. In addition, a series of differentially expressed genes between the D30FL and Ori groups could be mapped to corresponding gene families (Figure S4). This un-veiled that all differentially expressed genes in the ribosomal protein (RP) and solute carrier (SLC) families showed flexible change trends in the presence of the artificial restocking practice, which suggested that *Huso dauricus* might activate a spontaneous physiological regulatory response to adapt to the natural habitat through synergistic effects within the SLC and RP gene families.

### 2.7. Effects of Stock Enhancement on the Enteric FoxO1 Gene Expression Trend in Kaluga Sturgeon

Based on the transcriptome project for intestine tissue, it was demonstrated that the FoxO signaling pathway was involved in metabolism regulation activity during the artificial restocking period. According to the FPKM values provided by the differentially expressed gene filtration analysis, the transcription levels of the FoxO1 gene (Gene ID: TRINITY_DN22802_c0_g1_i1_1) on the 0th, 14th, and 30th d were 0.4, 52.2, and 188.4, respectively. Therefore, the FoxO1 protein expression level in gut tissue was also measured using an immunohistochemical technique (Figure S5). It was displayed that the FoxO1 protein level in the intestine continuously rose with a time-dependent effect, which suggested that re-wilding activated the intestinal FoxO1 protein expression to meet the physiological requirements for the wild external environment.

### 2.8. Effects of Stock Enhancement on Gut Histomorphological Changes in Kaluga Sturgeon

As shown in Figure S6a, the gut tissue in the Ori group displayed no apparent pathological characteristics. Lamina propria cells were arranged in alignment, and various cell types were observed. Meanwhile, the intestinal villus structure was nearly intact and had a tight link with the mucous lamina propria. In contrast, the mucosal epithelium of the intestinal villus on the 7th d of stock enhancement was broken, and a mass of shed epithelial cells was observed (Figure S6b,c). Dilated blood vessels were also recognized in the muscular layer region; myocytes displayed a loose arrangement trend, and some necrotic cell fragments were also observed (Figure S6b). As expected, only some shed intestinal epithelial cells could be found in the gut tissue on the 14th d, while the histological structures of the intestinal villus were still somewhat blurry (Figure S6d,e). Surprisingly, it was found that nearly no visible pathological injury except for a few shed mucosal epithelial cells, could be observed in the intestinal tract after 30 d of re-wilding (Figure S6f).

## 3. Discussion

The external manifestation of wildlife is usually used to assess its physiological adaptability to an environment [6,10,21]. Direct stock enhancement significantly increased the time that was taken to feed successfully for *Acipenser oxyrinchus* [6]. Interestingly, it was found that the gut content weight on the 7th, 14th, and 30th days of the restocking period was lower than that in the Ori group. Similarly, adequate feed debris could be found in all individuals of the Ori group, while nearly no residual food fragments were found in the D7FL group, which indicated that *Huso dauricus* could not capture live food alone in the natural habitat because of the stable hatchery rearing model [21]. In addition, although *Huso dauricus* could prey on various live aquatic organisms (such as small fish and invertebrates) after a 14 d and 30 d reintroduction period, the gut content weight was still lower than that in the Ori group. It could be inferred that *Huso dauricus* can only obtain limited food resources in the wild after restocking in a natural river environment [6]. Our results also confirmed that the sturgeon did not obtain food within the first 7 d of the restocking period, which promoted excessive fat consumption, thus inducing a decline in body weight. Subsequently, *Huso dauricus* urgently captured limited prey resources to supplement the exhausted energy, which promoted compensatory fat storage.

Meanwhile, a series of pathological injuries, including shed mucosal epithelial cells, debris and residue of necrotic cells, and dilated blood vessels, were also observed in the gut 7 d after restocking, while these characteristics were gradually alleviated from the 14th to the 30th d of the rewilding period. These results might suggest that the initial stock enhancement period (within 7 days) induced intestinal tissue injury due to available food deficiency and external environment stress [6]. Subsequently, *Huso dauricus* could capture some prey to meet its essential nutrition requirements, which repaired the damaged gut tissue; this repair was accompanied by the relief of the stress condition. Consistent with previous reports, adverse environmental conditions can cause serious tissue injury, disturbing normal functional homeostasis [31].

Serum biochemical parameters are associated with internal physiological response under environmental stress [33]. The cortisol level represents the stress status when an animal faces an external environmental stimulus [34]. A previous study claimed that long-term repeated musical stimulation significantly decreased the serum and salivary cortisol levels in 40-day-old hybrid growing piglets [35]. In the current research, the serum cortisol concentration of *Huso dauricus* first underwent a visible elevation within the initial 7 days and was then restored to the normal level from the 14th to the 30th d of artificial stock enhancement. Our results indicated that sudden transfer to the wild natural environment would accelerate *Huso dauricus* in developing a physiological stress status because of a series of features that are inadaptable to the natural habitat. Subsequently, *Huso dauricus* began to gradually adapt to the wild environment, which was accompanied by the restoration of autonomous predation and a capacity for the processing of external environmental challenges from the 14th d after restocking. Accordingly, it could be supposed that formal stock enhancement for *Huso dauricus* should be performed after 14 d of wilderness training because the 7th–14th d of the re-wilding period can be regarded as an adaptability recovery period for artificial reintroduction. In addition, a stress-regulation-related signaling pathway (renin-angiotensin system) and GO gene clusters (“lysosomal lumen” and “lysosome”) were also enriched between the D14FL and D30FL groups, indicating that the 14th–30th d might be a critical time point for alleviating stress status during stock enhancement. Normal liver functions are usually related to nutrition usage, immune defense, and environmental stress adaption in organisms [36]. Xenobiotic stress decreased the total protein and albumin levels and elevated the alanine aminotransferase and aspartate aminotransferase activity in the serum of female fattening pigs [37,38]. Similarly to these results, our study also found that the serum total protein level was decreased and began to be restored to the normal level on the 14th d of re-wilding. In parallel, the aspartate aminotransferase and alanine aminotransferase activity on the 30th d of stock enhancement was statistically significantly different from that in the Ori group. Total protein is mainly synthesized by the liver and can be regarded as an important biomarker for hepatic synthesis capacity [39]. In addition, aspartate aminotransferase and alanine aminotransferase are involved in various metabolic activities and are reliable indicators for evaluating comprehensive liver function conditions in the presence of external stimuli [40]. Therefore, our results suggested that initial stock enhancement (within 7 days) caused physiological stress conditions, which directly affected the hepatic synthesis potential and, thus, disturbed the transamination process between amino acid and α-keto acid. Interestingly, we also found that typical transaminase activity was significantly increased 30 d after artificial stock enhancement, which might be attributed to *Huso dauricus* altering the hepatic metabolism mode after transfer to the wild habitat in response to the complex conditions in the natural river. Ion homeostasis also plays regulatory roles in the maintenance of normal physiological activities and directly reflects the health condition of wildlife. It was demonstrated that the blood potassium and phosphorus distribution was changed after artificial stock enhancement, which indicated that the restocking project affected the electrolyte balance mode of *Huso dauricus* to meet the metabolic requirements in the wild environment. Previous research also reported that environmental stress sources obviously decreased the iron and copper contents in mammals to reconstruct a physiological metabolism mode [38]. In addition, other biochemical indexes, including myocardial enzymes, renal function parameters, and blood lipid levels, displayed similar changes, suggesting that reintroduction to a natural habitat affected the normal functions of multiple organs in *Huso dauricus* to adapt to the external complex environment by modulating the comprehensive metabolic interaction network.

The intestinal epithelial barrier is a protective structure that prevents pathogens’ invasion of the lumen and separates the intestinal cavity from the external environment. In this process, tight-junction proteins are pivotal components for maintaining tight connections between single cells and regulating the gut’s permeability [41]. Upon exposure to an environment rich in heavy metals, ZO-1 and Occludin protein expression was significantly inhibited in the gut tissue of wild *Hipposideros armiger* bats [23]. It might be implied that adverse environmental stress disturbs the intestinal tight-junction conditions. The present research also revealed that artificial stock enhancement downregulated the expression trends of tight-junction proteins in the gut of *Huso dauricus* within 7 days and then gradually restored them to the normal levels from the 14th to the 30th d. These results indicated that the initial reintroduction process decreased the intestinal tight junction to expand the cellular spaces and raised permeability to harmful substances, which induced intestinal stress conditions and obvious gut tissue injuries. With the gradual process of adaptation to wild environments, restored intestinal physiological homeostasis promoted the expression of tight-junction proteins to establish a blocked gut environment [41].

Artificial stock enhancement obviously affected the colonization conditions of gut microbial taxa in sturgeon [1]. Thus far, one study has demonstrated that re-wilding affects the species diversity of micro-organism taxa in a series of amphibian species, including *Atelopus varius* [12]. One ecological monitoring study found that the hatchery rearing mode obviously alters the α-diversity of skin bacterial communities in *Centropomus undecimalis* and enhances the evenness and richness [27]. Our study also demonstrated that the Shannon and Simpson indexes of gut bacterial communities in Kaluga sturgeon were significantly elevated 14 d after restocking. This could be attributed to *Huso dauricus* requiring the establishment of a reconstituted microbial structure to adapt to the challenge of the wild environment after a physiological stress period (7 days) [42]. Some reports suggested that high α-diversity would counterbalance the negative impacts of a captive breeding mode [43]. Consistent with our results, *Huso dauricus* did not adequately adapt to the wild habitat until the 14th d of the period. It was also confirmed that 14 d of wilderness training may be essential for artificial stock enhancement for wild *Huso dauricus*. In addition, the β-diversity analysis revealed that the intestinal microbiota among the four groups tended to fully separate from each other, and greater distance was displayed between the Ori and D7FL groups, indicating that the gut bacterial composition suffered from drastic reconstruction during the stock enhancement period, especially on the 7th d due to the physiological stress conditions in the *Huso dauricus* population caused by inadaptability to the wild environment. Similarly to our study, a previous report found that the re-wilding process also altered the β-diversity indexes of *Centropomus undecimalis* based on a multidimensional scaling analysis [27]. Various microbial populations could be embedded into the surfaces of self-produced polymeric matrices to compose a biofilm structure, which would enhance the gut microbial taxon diversity and maintain colonization stability under adverse environmental conditions [44]. It could be supposed that stock enhancement might regulate the interaction mode of complex polymeric matrices to evoke a change in the community diversity of gut bacteria. In addition, according to the insights into the microbial structure, the most suitable adaptation period during re-wilding practices might be species-specific. An existing report demonstrated that *Cervus nippon* required nearly 53–60 d for gut microbial diversity, while amphibians only needed 27 d to adapt to the wild environment [12,43]. Gut microbes have various detailed functions that modulate physiological metabolic processes in the host. Our results demonstrated that the relative abundance of the Firmicutes phylum was first elevated on the 7th d and then gradually declined nearly to the original level from the 14th to the 30th d of stock enhancement. In addition, Firmicutes became one of the prominent taxa after 7 d of reintroduction; subsequently, Proteobacteria became the dominant phylum again from the 14th d to the 30th d. Firmicutes protects the intestinal tract from a high-fiber diet by promoting the transfer of fiber components in the gut and secreting a series of active hydrolase substances that could degrade fiber [45]. *Huso dauricus* could not capture live prey to meet the requirements for nutrition within the first 7 days; therefore, they might have been forced to gnaw on submerged, withered leaves in a hungry state. In this process, colonization by Firmicutes taxa promoted the digestion of fiber, which was beneficial for *Huso dauricus* by allowing it to absorb the limited energetic substances during the initial stress period. The Firmicutes phylum is also positively related to the development of infectious disease [13]. It is speculated that the elevated abundance of Firmicutes would cause *Huso dauricus* to be susceptible to infectious disease during the stress period (initial 7 days). Therefore, it is inopportune to directly restock *Huso dauricus* in wild habitats before the peak value of Firmicutes has been reached. In addition, we found that the Proteobacteria phylum had the opposite change trend to that of Firmicutes. The Proteobacteria phylum is usually positively associated with the level of intake of crude protein [46]. Obviously, the relative abundance of Proteobacteria declined within 7 d because *Huso dauricus* could only intake some high-fiber withered leaves; then, its colonization was promoted when *Huso dauricus* could autonomously capture live aquatic organisms to supplement its protein requirements after the 14th d of stock enhancement. The Pseudomonadaceae family is usually associated with the maintenance of physiological balance in the intestinal micro-environment and can trigger the expression of encoding genes related to cyanide degradation [47]. Moreover, recent research also confirmed that bacterial communities that belong to the Pseudomonadaceae family carry various mercury-associated resistance genes. Therefore, it might contribute to tolerance of exogenous heavy metal elements in vertebrates, including birds and aquatic organisms [48]. Our results suggested that the Pseudomonadaceae family was the dominant biomarker member on the 14th d of stock enhancement, suggesting that *Huso dauricus* aimed to detoxify itself from aquatic xenobiotics (such as heavy metal residues and cyanide substances from geochemical cycling) in the wild habitat by promoting the colonization by the Pseudomonadaceae family. In addition, it might have played an essential role in the establishment of adaptability to the natural environment because it was confirmed that *Huso dauricus* had eliminated its physiological stress status after the 14th d of the restocking period. Meanwhile, some environmental microbe species that usually exist in natural waters (such as the *Phreatobacter* genus) could also be identified in the taxa of the intestinal microbiota, suggesting that *Huso dauricus* had microbial communication with the external environment [49]. The re-wilding process affects the microbial functions in the intestines of wildlife, and it is involved in regulating the physiological activity of the host [43]. KEGG enrichment analysis revealed that the relative proportions of four differential pathways displayed dynamic volatility over the 30 d of stock enhancement for *Huso dauricus*. This indicated that the endocrine system modulated the environmental adaption to the wild habitat through the strict interaction effects of various hormones, and the establishment of adaptability to the external environment might have depended on a microbial secondary metabolite that controlled the co-transport process of a biofilm system. Previous investigations also found that these predicted functions of gut microbes were also significantly enriched in the context of re-wilding practices for *Centropomus undecimalis* (common snook) [27] and *Panthera tigris altaica* (Amur tiger) [13].

Changes in gene expression profiling might be involved in the process of adaption to the external environment. The FoxO1 gene is involved in the regulatory mechanism of the cell cycle, programmed death, anti-oxidant response, and immune defense [50]. Our results showed that the transcription/protein levels of FoxO1 were continuously elevated from the 0th d to the 30th d, and the FoxO signaling pathway was enriched based on the KEGG analysis. Stock enhancement might cause *Huso dauricus* to express the FoxO1 protein with an obvious time-dependent effect, which would enhance the anti-oxidant capacity and anti-pathogen defense in the wild habitat. In the natural environment, animals would initiate a self-adaption strategy and adjust their metabolic process to meet the physiological requirements. A previous study also proved that environmental stress elicited the overexpression of the FoxO1 gene [50]. Interestingly, the transcriptome project enriched immune-related genes and GO term clusters in the gut tissue. Our study found that the expression levels of IL-1β and MMP9 were durably upregulated within 30 d of stock enhancement. IL-1β usually participates in the induction of cytokines/chemokines and triggers a cascaded immune response to exogenous stimuli [51]. MMP9, which mainly derives from neutrophils, is involved in the development of inflammation and tissue injury repair [52]. Similar research also found that the MMP9 gene was associated with antagonistic roles in environmental stress [53]. These results demonstrated that *Huso dauricus* enhanced its immune defense capacity after artificial restocking to resist precipitous environmental conditions in the wild habitat. In the present investigation, the expression trend of the CYP family remained at relatively stable levels on the 14th d, and the “Drug metabolism-cytochrome P450” signaling pathway was also enriched between the D30FL and Ori groups. Activation of the CYP family triggers the accumulation of reactive oxygen species in mitochondria, which accelerates the occurrence of the oxidative stress response [54]. In our study, CYP expression remained at a relatively stable level in the gut after the 14th d of stock enhancement and was lower than that in the Ori group, suggesting that *Huso dauricus* had preliminarily adapted to the external environment with the restoration of its foraging capacity from the 14th d; thus, the oxidative stress status in the gut might have been kept low. In addition, *Huso dauricus* might decrease its anti-oxidant defense status to economize limited energy consumption, thus assigning more energy to essential metabolic activity when facing a comprehensively wild environment. Interestingly, the dynamic expression of ATP-metabolism-related genes identified in the present research also demonstrated that the synthesis/degradation of energetic substances is involved in the process of adaption to wild habitats in *Huso dauricus* during an artificial stock enhancement period [23]. Consistently with these results, “Fatty acid degradation”, “Glycolysis/Gluconeogenesis”, and “Oxidative phosphorylation” were also enriched on the 14th d and 30th d, which suggested that *Huso dauricus* might maintain normal energy requirements in the wild habitat by regulating the lipid/carbohydrate metabolism and electron transport in the respiratory chain. In parallel, some studies confirmed that the SLC gene family participates in the physiological regulation process under stress conditions [23]. The SLC gene family can transport mitochondrial ATP to the cytoplasm region and has been confirmed to be associated with immune defense, anti-oxidant response, essential substance transport, and programmed cell death induction [55]. For example, the SLC22/26 gene families play carrier roles in the cellular anion transport process, while the SLC6 gene family can be regarded as a critical transporter of amino acids and is involved in a series of inflammation-development-related signaling pathways [56]. Our transcriptomics results also revealed that a series of SLC-family-related genes participated in the physiological regulatory response during the 30 d stock enhancement period. Hence, this might suggest that the intestine of *Huso dauricus* modulated the transport process of various necessary nutritional substances (e.g., amino acids and organic ions) to meet the requirements for environmental adaptation to the wild habitat by regulating the expression patterns of the SLC gene family. The RP gene family, which plays normal regulatory functions in the cytoplasm, is mainly responsible for the biological synthesis of ribosomal subunits and the physiological defense response to exogenetic stress status [57]. The RPL gene cluster is regarded as a crosstalk bridge between ribosomes and RNA, and it affects intracellular protein translation, cell death activation, and cellular source usage [57]. Our study also found that various RPL genes in the intestine were downregulated after restocking in the wild habitat. Therefore, *Huso dauricus* primarily altered the expression levels of RPL genes in the intestine to regulate the protein synthesis process and complex physiological functions, which could be beneficial in maintaining intestinal homeostasis during the period of adaptation to the external environment. Taking these results together, fish species might adapt to alterations in the external environment through “gene–microbe”-interaction-related regulation mechanisms [6,48,58,59].

## 4. Materials and Methods

### 4.1. Experimental Design and Sample Collection

**Figure 7:**
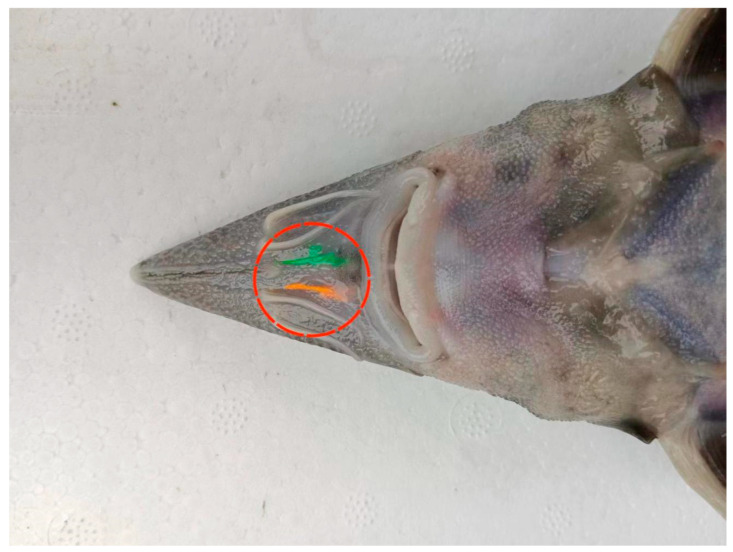
Characteristic snout of *Huso dauricus*. The red circle represents the fluorescent signature that was injected before artificial stock enhancement.

On 9 May 2023, nearly ten thousand Kaluga sturgeon oosperms with purebred lineage were bred in professional hatching pools (Harbin, China) at 18.9–19.6 °C. After the rupture of membranes, healthy larval fish were selected and cultured in the following suitable environment: breeding temperature: 14.8–16.2 °C, breeding density: 500 individuals/m^2^, pH value: 6.6–8.3, dissolved oxygen: more than 6.0 mg/L. The water quality was monitored to maintain a lack of pollution and to ensure that the water was transparent. During the early larval stage, all individuals were transferred to automatically temperature-controlled rearing pools and fed with live fish bait (*Limnodrilus*). After the early growth stage, Kaluga sturgeons were fed with commercial feed (ALLER AQUA, Harbin, China) that was consistent with the basic nutrition requirements of juvenile fish (Table S5). All subjects were fed four times per day based on 5% of their body weight. The external breeding environment parameters were as follows: breeding temperature: 15.1–16.3 °C, breeding density: 150 individuals/m^2^, pH value: 6.3–7.5, water velocity: 0.22 m/s, nitrite nitrogen: 0.002 mg/L. The aquaculture water in the artificial breeding stage was stored in aeration pools for 24 h to remove chlorine residue and was supplemented with dissolved oxygen. On October 6th, one thousand Kaluga sturgeons with a similar body weight and length were released into the Songhua River (119°52′–132°31′ E,11°12′–51°38′ N) after a 3 h environmental adaptation period to eliminate the physiological response caused by transport stress. Before wild reintroduction to the river, non-toxic fluorescent dye was injected into the maxillary surface of the Kaluga sturgeon (Figure 7), which was beneficial for catching them again. On the 0th day (Ori), 7th day (D7FL), 14th day (D14FL), and 30th day (D30FL), Kaluga sturgeon individuals were recaptured randomly using seine nets within a 10-km radius. In our study, the Ori group was regarded as the control group because all individuals in this group were sampled before artificial stock enhancement in the Songhua River. All five subject individuals were anesthetized via the intake of tricaine methane sulfonate (M14788, ABMOLE, Shanghai, China) after their body weight and length were recorded. Blood samples were collected from the caudal vein with 1 mL injection syringes and then stored in a sterile blood-collecting vessel with a coagulant to separate the serum at 4 °C and 12,500 rpm. Subsequently, each intestinal segment was ligated using nylon wire, and then gut content samples were collected from the hindgut using sterile scalpel blades. Serum, hindgut tissue, and gut content samples were stored in RNase-free centrifuge tubes for the following molecular biological detection and morphological observation. Additionally, residual food debris in the stomach was fixed in anhydrous ethanol to identify the feeding habits of the Kaluga sturgeon during the restocking period. All operation procedures were in accordance with the guidelines for the care and use of experimental animals that were proposed by Heilongjiang River Fisheries Research Institute (20230925-001).

### 4.2. Paraffin Section Preparation and Immunohistochemistry Staining

Fresh gut tissue was washed with precooled 0.9% saline solution and then fixed in Bouin’s fixative reagent (BL-G016, SBJBIO, Nanjing, China) for 10 h to maintain tissue morphology. Then, tissue blocks were dehydrated in a gradient concentration of ethanol solutions and submerged in xylene for 15 min for hyalinizing. Subsequently, tissue blocks were put into melted paraffin and cooled at −20 °C for 1 h; then, they were cut into 3 µm tissue sections with a pathology slicer (RM-2016, LEICA, Wetzlar, Germany). After dewaxing in a series of transparent dewaxing liquids (G1128, SERVICEBIO, Wuhan, China), the tissue sections were immersed in anhydrous ethanol and rinsed with distilled water to hydrate adequately. A citric acid antigen repair solution (pH = 6.0) was added into sections under heat induction conditions to restore the antigenic determinant. In the dark, a hydrogen peroxide solution was used to inactivate endogenous peroxidase, and the tissue sections were covered with 3% bovine serum albumin to block for 30 min. Then, the tissue sections were incubated with the primary antibodies (SIGNALWAY ANTIBODY, College Park, MD, USA; Claudin1, Zonula occludens (ZO-1), and occludin, 1:150 dilution; FoxO1, 1:250 dilution) for 18 h and the secondary antibody (goat anti-rabbit IgG, 1:350) for 85 min at 4 °C. Subsequently, 3, 3′-diaminobenzidine dye liquor (GK800511, GENETECH, Shanghai, China) was used to dye the target proteins while hematoxylin was added on the section surface to re-dye the cell nuclei. After the paraffin sections were dehydrated again and sealed, a digital microscope system (E-100, NIKON, Tokyo, Japan) was utilized to take photos for each sample three times. Cell nuclei were shown in blue, while positive expression areas were stained brown.

### 4.3. Blood Parameter Measurement

Whole blood samples were collected from the caudal vein and then stored in ice-bath conditions to prevent the degradation of bioactive substances. After the standard detection parameters were set, the test samples were added to liquid transfer tanks, and hematology indexes (cortisol, blood glucose, blood potassium, blood sodium, blood chlorine, blood calcium, blood phosphorus, blood magnesium, cholesterol, triglyceride, high-density lipoprotein cholesterol, low-density lipoprotein cholesterol, creatine kinase, creatine kinase-MB, α-hydroxybutyrate dehydrogenase, carbon dioxide combining power, alanine aminotransferase, aspartate aminotransferase, alkaline phosphatase, lactate dehydrogenase, albumin, and total protein) were measured using an automatic biochemical analyzer (DXC-600, BECKMAN COULTER, Indianapolis, IN, USA). In addition, typical electrolyte ion concentrations in serum were also detected based on the membrane electrode method using an automatic electrode analyzer (EasyLyte Plus, MEDICA, Derwood, MD, USA).

### 4.4. Non-Reference-Genome-Based Transcriptome Sequencing

First, 150 mg of fresh intestinal tissues (Ori, D14FL, and D30FL) was mixed with TRIzol reagent (15596026CN, INVITROGEN, Carlsbad, CA, USA) to extract the total RNA samples, the concentration and purity of which were identified using a spectrophotometer (NanoDrop 2000, THERMO, Waltham, MA, USA). RNA samples with an RNA integrity number (RIN) value of more than 7.0 were used to build a sequencing library using a VAHTS Universal V6 RNA-seq Library Prep kit (NRM-604, VAZYME, Nanjing, China). After the quality control for the original transcriptome library, a paired-end sequencing project was run based on the Novaseq 6000 platform (ILLUMINA, San Diego, CA, USA), which generated 150 bp fragment sequences to implement the downstream analysis. Detailed data processing and a bioinformatics analysis procedure were provided by OE BIOTECH (Shanghai, China). Low-quality reads, adaptors, and specific reads with poly N were removed using the TRIMMOMATIC tool to obtain clean reads that were spliced to transcript sequences using the De novo method in the TRINITY program. All clean data utilized for the following bioinformatic analysis process were uploaded to the public Gene Expression Omnibus genome repository (https://www.ncbi.nlm.nih.gov/geo/query/acc.cgi?acc=GSE266327, accessed on 1 May 2024). The longest transcript was regarded as one UniGene. The fragments per kilobase of transcript per million mapped reads (FPKM) value for each UniGene was calculated using the eXpress software (Version 1.5.0), while BOWTIE2 was used to identify the number of reads that could be mapped to the UniGene in each sample. After the standardization process of the FPKM values, DESeq was utilized to calculate the fold change and *p*-value based on the NBINOM TEST function. Each UniGene with *p* < 0.05 and |log_2_ fold change| of more than 1.0 was regarded as a differentially expressed gene. All differentially expressed genes were subjected to hierarchical cluster analysis to display the gene expression mode between treatment groups. In addition, specific gene family members were filtered and visualized with heatmaps. Each differentially expressed UniGene (E value was less than 10^−5^) was mapped to Gene Ontology (GO) terms and Kyoto Encyclopedia of Genes and Genomes (KEGG) signaling pathways based on the annotation results from the SWISSPROT database.

### 4.5. 16S Ribosomal DNA Amplicon Sequencing

A commercial DNA LQ kit (D6356-02, MAGEN, Guangzhou, China) was used to extract the genomic DNA of the bacterial population from the intestinal content of the Kaluga sturgeon. After the DNA concentration and purity were measured via agarose gel electrophoresis and spectrophotometry, the variable V3-V4 region was amplified using specific primers with a barcode sequence (343F: TACGGRAGGCAGCAG, 798R: AGGGTATCTAATCCT) and Tks Gflex DNA Polymerase (R060B, TAKARA, San Jose, CA, USA). The details of the procedure can be described as follows: 94 °C, 5 min, 1 cycle; [94 °C, 30 s, 56 °C, 25 s, 72 °C, 25 s], 35 cycle; 72 °C, 5 min, 1 cycle. The purified product was used for the secondary polymerase chain reaction system, and the end product was purified using AMPure XP beads. Paired-end (2 × 250 bp) sequencing was performed based on the Hiseq 2500 platform (ILLUMINA, San Diego, CA, USA) after the concentration of the final product was detected. The CUTADAPT software (Version 1.9) was used to cut the barcode sequence off of the raw tags, and then quality filtration, denoising, splicing, and chimera elimination were accomplished using the DADA2 tool to generate an amplicon sequence variant (ASV) feature sequence/table. Representative tags for each amplicon sequence variant were selected according to the default parameters provided in QIIME2 and then blasted with the SILVA138 database. In addition, the α-diversity and β-diversity indexes were calculated based on the standard equations, and significant differences between treatment groups were verified using an independent-sample *t*-test and the weighted Unifrac distance algorithm. For the species annotation process, the q2-feature-classifier software was adopted to map the amplicon sequence variant features to the corresponding taxonomic classifications. Meanwhile, significance analysis of the differences in the abundance of bacterial populations was performed based on the one-way analysis of variance method in the R package environment. Finally, the PICRUSt2(2.3.0b0) tool was used to predict the relative abundance of gene functions in the known microbial community based on the public KEGG repository, and the Kruskal–Wallis test was applied to compare the significance of the differences among these four groups.

### 4.6. Histopathological Observation for Gut Tissue

After all Kaluga sturgeon individuals were slaughtered, the abdominal cavity was dissected, and then gut tissues were quickly removed, followed by fixing in 10% formalin solution (F8775, SIGMA-ALDRICH, Saint Louis, MO, USA) for 30 h. Subsequently, intestinal tissues were immersed in a concentration gradient of ethyl alcohol using an automatic dehydrator (Donatello, DIAPATH, Venice, Italy) for the dehydration procedure and then were embedded into melted paraffin wax (JB-P5, JUNJIE, Wuhan, China) to prepare for the gut tissue slices. After dewaxing for 55 min, tissue sections were dyed using a hematoxylin and eosin staining solution for 4 min. Finally, the tissue slices were sealed with neutral gum (10004160, SINOPHARM, Beijing, China) after hyalinizing and were observed using a digital optical microscope (DM3000, LEICA, Wetzlar, Germany). In addition, intestinal injury pathology was analyzed using the open-access QuPath software (Version 0.5.1, Belfast, Northern Ireland).

### 4.7. Data Processing and Statistical Analysis

The data analysis process was implemented using the IBM Statistical Product and Service Solutions Statistics software (Version 27.0, Chicago, USA). All data used in the current investigation are shown as the mean ± standard deviation and are reported to two decimal places. Before the variance analysis was performed, normal distribution characteristics and the homogeneity of variance were verified based on the Shapiro–Wilk test and the Levene method. Subsequently, a one-way analysis of variance with the least significant difference method was performed to compare the significance of the differences between treatment groups (Ori, D7FL, D14FL, and D30FL). A difference was regarded as statistically significant when the *p*-value was less than 0.05.

During the data processing for multi-omics analysis, program scripts referring to standard bioinformatic analysis methods and graph visualization processes were completed using corresponding tools in the R package. Specifically, α-diversity indexes were calculated according to the “alpha-diversity py” command, and an independent-sample t-test was used to examine the statistical significance among four groups with the IBM Statistical Product and Service Solutions Statistics software (Version 27.0, Chicago, IL, USA). Non-metric multidimensional scaling (NMDS) analysis was performed using the R package “vegan” to obtain the stress coefficient, and then the “aov” package was selected to implement the difference significance analysis (one-way analysis of variance) for the microbial taxon composition among the four groups. Subsequently, Tukey’s post hoc test was carried out using the “multcomp” package. Meanwhile, linear discriminant effect size (LEfSe) analysis was completed for these four groups using the “microeco” tool. Data analysis files that were generated in the microbiome and transcriptome analysis process were visualized using the “ggplot2” package. The detailed data analysis for multi-omics was supported by the Omics Cloud platform (OE BIOTECH, Shanghai, China).

## 5. Conclusions

Our investigation revealed the dynamic impacts of artificial stock enhancement on *Huso dauricus* on the molecular level for the first time. Multiple techniques demonstrated that *Huso dauricus* was in physiological stress conditions for the initial 7 d of the reintroduction period and then gradually adapted to the wild environment by the 14th d of the restocking practice. Meanwhile, our results suggest that the dynamic adaptability to the natural habitat could be attributed to the comprehensive volatility of gut microbial taxa and gene expression profiling. Our study confirms that wilderness training is essential for *Huso dauricus* to establish adaptability to natural habitats and sheds light on the further exploration of appropriate stock enhancement strategies for wild sturgeon.

## Data Availability

The original data used and generated in the present research project are available from the corresponding authors upon request, with a completed Data Transfer Agreement. Raw sequencing data can be downloaded from the National Center for Biotechnology Information repository (https://www.ncbi.nlm.nih.gov/geo/query/acc.cgi?acc=GSE266327, accessed on 1 May 2024).

## Supplementary Materials

The following supporting information can be downloaded at: https://www.mdpi.com/article/10.3390/ijms26041480/s1.
