# Supplementary material for: Dynamic Impacts of Stock Enhancement on Kaluga Sturgeon (Huso dauricus): Novel Conservation Strategy Insights from the Gut Microbe Composition and Gene Expression Mode
Source: Int J Mol Sci. 2025 Feb 10;26(4):1480. doi: 10.3390/ijms26041480 (PMC11855664; doi:10.3390/ijms26041480)
Supplement: Supplementary file 1 [file ijms-26-01480-s001.zip › ijms-3387854-supplementary.pdf]

Table S1 Growth performance and blood parameters in kaluga sturgeon

	Ori	D7FL	D14FL	D30FL
Body length (cm)	25.68±2.33	23.46±2.88	27.58±3.58	28.43±1.49
Body weight (g)	61.47±12.42	48.32±15.36	72.20±25.00	81.54±12.90
Body weight / body length	2.38±0.29^{ab}	2.03±0.40^{b}	2.57±0.54^{a}	3.04±0.17^{a}
Gut content weight (g)	0.88±0.47^{a}	0.36±0.19^{b}	0.41±0.46^{ab}	0.41±0.32^{ab}
Cortisol (μg/dL)	7.78±2.20^{b}	30.65±7.40^{a}	15.19±7.03^{b}	15.40±4.35^{b}
Blood glucose (mmol/L)	5.22±1.37^{a}	6.41±0.83^{a}	9.24±2.28^{a}	6.44±3.68^{a}
Blood potassium (mmol/L)	4.32±0.61^{a}	2.32±0.38^{b}	2.54±0.25^{b}	2.63±0.59^{b}
Blood sodium (mmol/L)	124.44±4.63^{a}	126.78±8.55^{a}	131.34±1.99^{a}	119.95±19.02^{a}
Blood chlorine (mmol/L)	109.00±4.43^{a}	110.68±9.57^{a}	114.90±2.91^{a}	106.55±15.20^{a}
Blood calcium (mmol/L)	1.73±0.56^{a}	1.76±0.88^{a}	1.75±0.71^{a}	1.72±0.49^{a}
Blood phosphorus (mmol/L)	4.21±0.57^{a}	3.05±0.44^{b}	2.80±0.11^{b}	3.48±1.51^{ab}
Blood magnesium (mmol/L)	0.88±0.15^{a}	0.80±0.08^{a}	0.92±0.09^{a}	0.93±0.21^{a}
Cholesterol (mmol/L)	1.05±0.13^{b}	1.54±0.59^{ab}	1.77±0.49^{a}	1.00±0.44^{b}
Triglyceride (mmol/L)	4.37±0.46^{a}	5.65±1.94^{a}	5.96±1.44^{a}	3.97±1.62^{a}
High density lipoprotein cholesterol (mmol/L)	0.13±0.02^{b}	0.20±0.03^{a}	0.24±0.04^{a}	0.20±0.03^{a}
Low density lipoprotein cholesterol (mmol/L)	0.48±0.15^{a}	0.77±0.37^{a}	0.81±0.26^{a}	0.49±0.21^{a}
Creatine kinase (U/L)	416.55±228.91^{b}	520.75±156.95^{b}	873.98±285.81^{ab}	1412.47±711.69^{a}
Creatine kinase-MB (U/L)	279.40±140.28^{a}	408.75±286.39^{a}	266.85±39.84^{a}	384.27±253.06^{a}
α-hydroxybutyrate dehydrogenase (U/L)	723.85±108.25^{a}	676.38±88.22^{a}	850.28±412.89^{a}	557.07±288.44^{a}
Carbondioxide combining power	6.29±0.64^{b}	5.21±1.38^{b}	9.23±1.33^{a}	5.56±0.82^{b}
Alanine aminotransferase (a*, U/L)	51.00±15.18^{b}	125.68±36.50^{a}	138.73±77.95^{a}	129.53±51.02^{a}

	Ori	D7FL	D14FL	D30FL
Aspartate aminotransferase (b*, U/L)	510.10±188.70^{b}	599.38±44.99^{b}	861.43±292.11^{ab}	883.57±85.06^{a}
b*/a*	9.20±5.45^{a}	6.45±1.05^{a}	6.06±1.85^{a}	9.79±2.65^{a}
Alkaline phosphatase (U/L)	331.53±89.45^{b}	192.46±23.00^{c}	388.58±126.29^{b}	811.60±249.62^{a}
Lactate dehydrogenase (U/L)	1118.66±140.19^{ab}	1152.23±107.63^{ab}	1238.15±500.35^{a}	743.10±175.58^{b}
Total protein (g/L)	6.64±1.89^{ab}	4.90±1.23^{b}	6.05±3.05^{ab}	8.63±2.23^{a}
Albumin (g/L)	3.40±1.50^{a}	3.88±0.44^{a}	4.30±0.94^{a}	4.80±2.35^{a}

Notes: Different lowercase on the upper right means statistical difference between treatment groups

Table S2 Feeding habits identification

Sample ID	Food composition
Ori1	Feed debris
Ori2	Feed debris
Ori3	Feed debris
Ori4	Feed debris
Ori5	Feed debris
D7FL1	Small-sized fish remains
D7FL2	--
D7FL3	--
D7FL4	--
D7FL5	--
D14FL1	Small-sized fish, mussel (*Anodonta*)
D14FL2	Small-sized fish
D14FL3	Pond crayfish (*Macrobrachium*)
D14FL4	Mussel (*Anodonta*)
D14FL5	Pond crayfish (*Macrobrachium*)
D30FL1	Mussel (*Anodonta*), river snail (*Viviparus*)
D30FL2	Small-sized fish, pond crayfish (*Macrobrachium*)
D30FL3	Small-sized fish, pond crayfish (*Macrobrachium*)
D30FL4	Pond crayfish (*Macrobrachium*), river snail (*Viviparus*)
D30FL5	Small-sized fish, mussel (*Anodonta*), pond crayfish (*Macrobrachium*)

Notes: --: not detectable

Table S3 Quality control for raw sequencing data

Sample number	Raw reads	Filtered	Denoised	Merged	Non_chimeric	Valid percentage (%)	ASV_counts	Total ASV
Ori1	79512	77392	77130	76491	76491	96.2	110	4399
Ori2	76915	74259	74025	73566	73566	95.65	77	
Ori3	78669	76801	76695	76427	76427	97.15	75	
Ori4	79197	76828	76597	76033	76033	96.0	108	
Ori5	78327	76048	75781	75352	75352	96.2	63	
D7FL1	77859	75460	75230	74325	74325	95.46	217	
D7FL2	79289	76703	76586	75848	75848	95.66	311	
D7FL3	79885	77928	77706	77030	77030	96.43	83	
D7FL4	78227	75879	75705	75385	75385	96.37	76	
D7FL5	78445	76014	75657	75162	75162	95.81	170	
D14FL1	78943	77069	76387	74441	74441	94.3	370	
D14FL2	78542	76496	76230	75202	75202	95.75	437	
D14FL3	79925	76566	76267	74725	74725	93.49	819	
D14FL4	80447	78018	77636	76369	76369	94.93	315	
D14FL5	78325	75938	75654	74773	74773	95.47	165	
D30FL1	79970	77954	77732	77230	77230	96.57	133	
D30FL2	80776	78251	77833	77025	77025	95.36	148	
D30FL3	79057	76563	76137	75202	75202	95.12	149	
D30FL4	78861	76884	76426	75385	75385	95.59	262	
D30FL5	78496	76472	76260	75809	75809	96.58	99	

Table S4 Summary for GO terms related to immune response

GO term	*P* value
response to cytokine	0.000255
defense response to Gram-positive bacterium	0.000379
C-C chemokine binding	0.003706
regulation of stress-activated MAPK cascade	0.003706
defense response to virus	0.005581
antibacterial humoral response	0.006199
inflammatory response	0.006451
response to virus	0.009043
immune response	0.009085
cellular response to tumor necrosis factor	0.011788
lymphocyte chemotaxis	0.028457
positive regulation of interleukin-6 secretion	0.028457
cytokine receptor activity	0.030879
positive regulation of innate immune response	0.030879
negative regulation of interleukin-8 secretion	0.031141
leukocyte migration involved in inflammatory response	0.031570
regulation of macrophage chemotaxis	0.031570
C-C chemokine receptor activity	0.031570
regulation of interleukin-1 beta production	0.031570
negative regulation of stress-activated MAPK cascade	0.031570
response to bacterium	0.036043
cytokine binding	0.039589
regulation of inflammatory response	0.040679
interleukin-6 receptor complex	0.045502
chemokine binding	0.045502
regulation of macrophage activation	0.045502
negative regulation of type I interferon production	0.047156
positive regulation of JAK-STAT cascade	0.047156
cellular response to granulocyte macrophage colony-stimulating factor stimulus	0.047156

Table S5 Detailed nutritional ingredient for juvenile kaluga sturgeon

Nutrient composition	Content
Crude protein	>49%
Crude fat	>12%
Crude fiber	<3%
Ash	<8%
Water content	<8.5%
Total phosphorus	<2%
Total calcium	>0.8%
Amino acid	>3%

Figure S1 Detailed taxa classification statistics

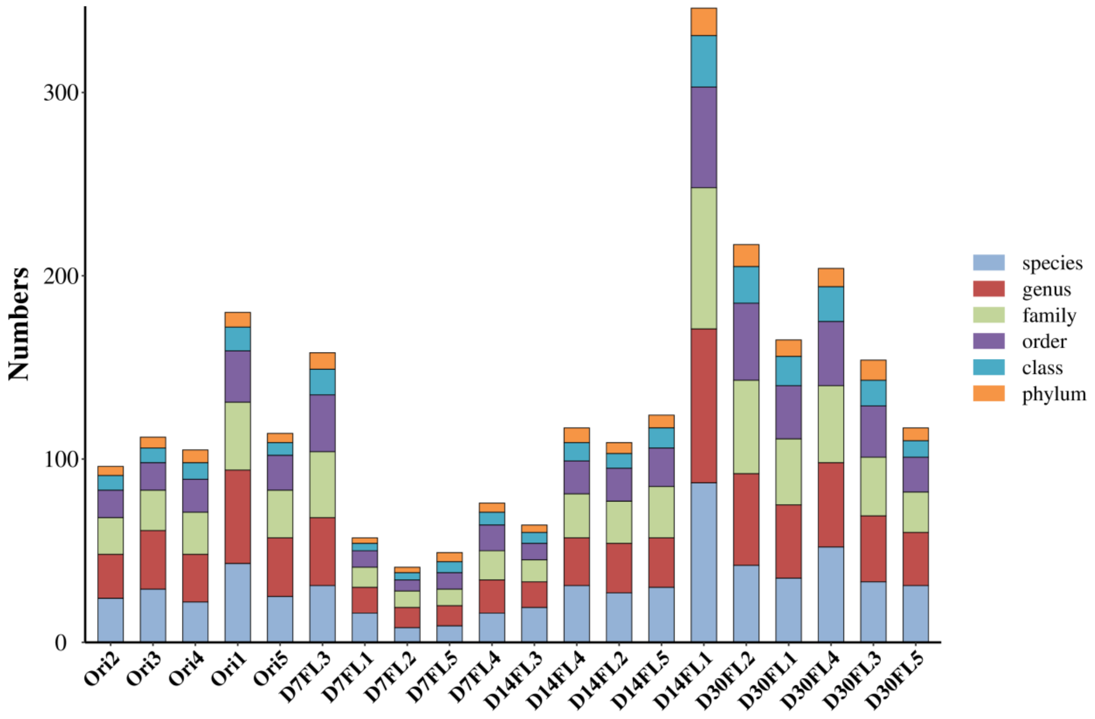

Notes: Abscissa axis represented the sample number. Ordinates axis represented the detailed species numbers for different classification levels.

Figure S2 Statistics for differentially expressed genes number

(a)

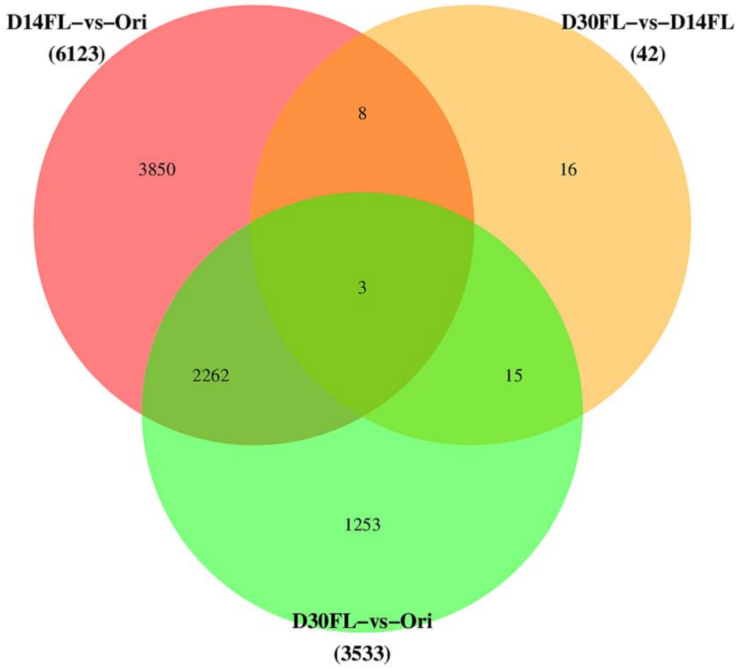

(b)

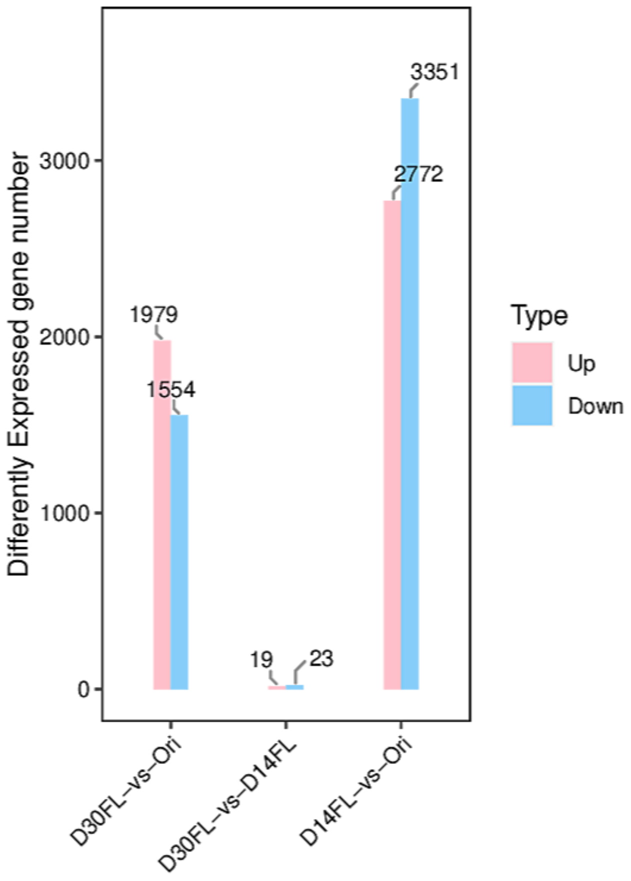

Notes: (a) Venn diagram for differentially expressed genes between different comparison groups. (b) Detailed up-regulated / down-regulated gene numbers in each comparison group.

Figure S3 Transcription levels of selected differentially expressed genes

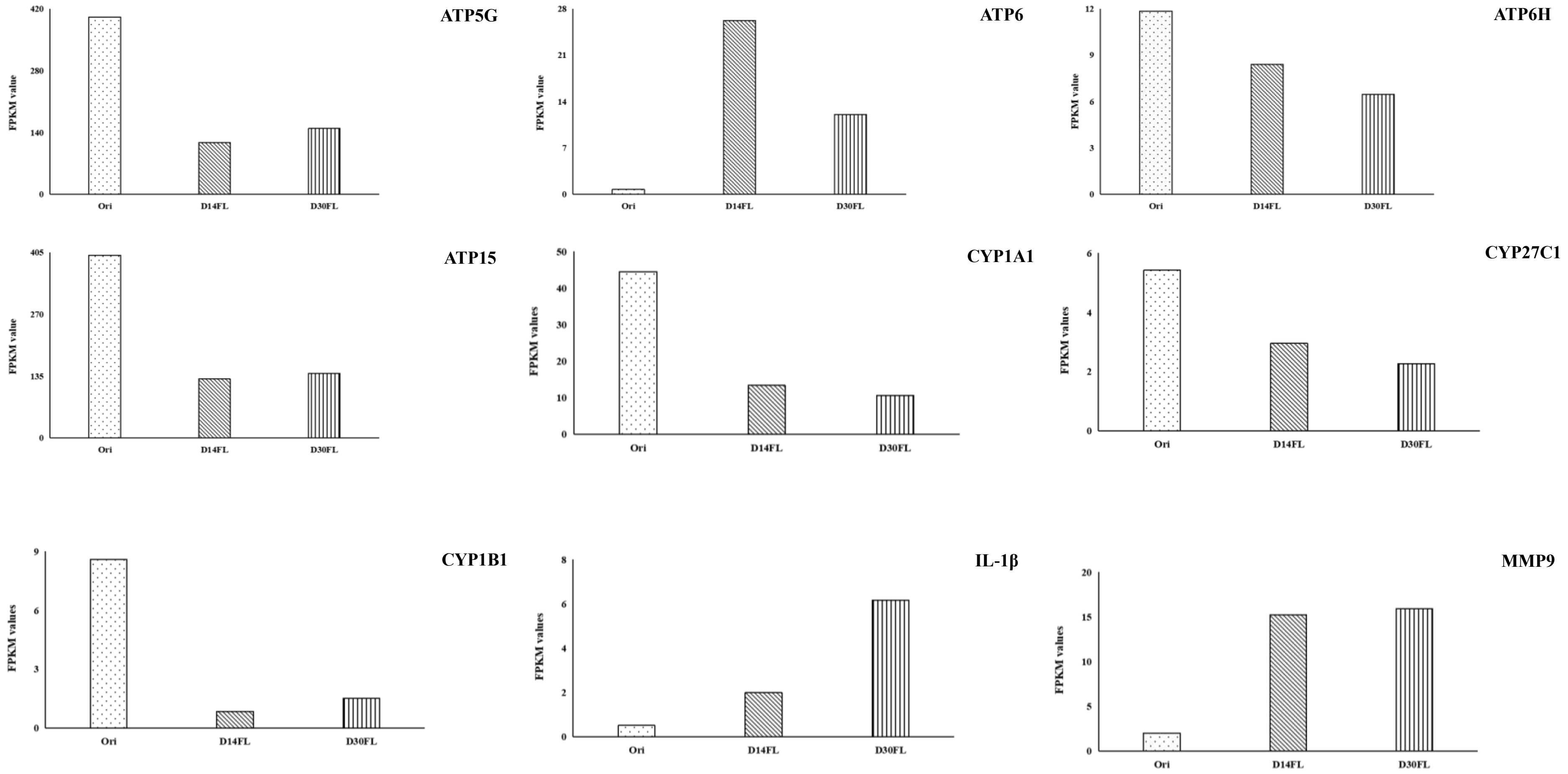

Figure S4 Heatmap for the SLC/RPL gene family selected from transcriptome profiling

(a)

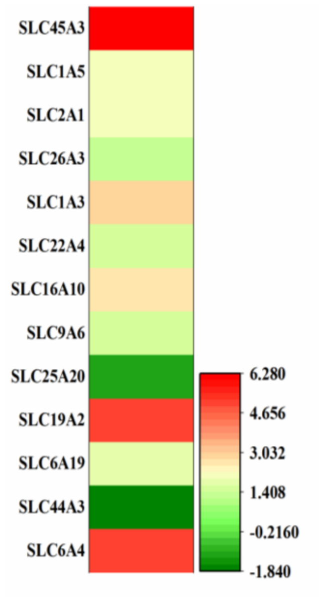

(b)

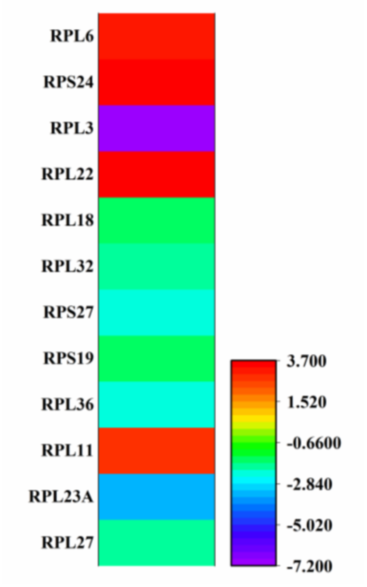

Notes: (a) SLC family. Color of heatmap represented the relative expression level ($\log_2$ [fold change] value) of each differentially expressed gene. Red: up-regulated trend; green: down-regulated trend. (b) RP family. Color of heatmap represented the relative expression level ($\log_2$ [fold change] value) of each differentially expressed gene. Color bar from red to purple indicated the transcription levels of RP family genes were down-regulated.

Figure S5 FoxO1 protein level in the intestine tract tissue of kaluga sturgeon

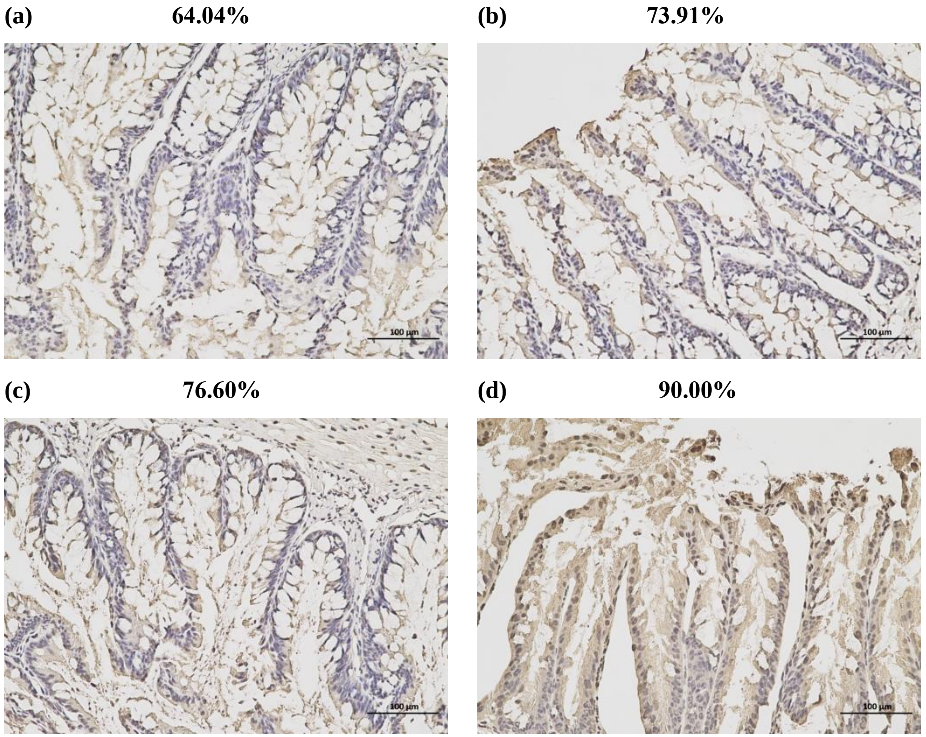

Notes: Each panel showed the intestinal FoxO1 protein expression level in (a) 0th d; (b) 7th d; (c) 14th d and (d) 30th d. Brown color indicated the positive expression area of FoxO1 protein while blue color indicated the cell nuclei (200×). Values in each panel represented the positive area ratio of FoxO1 protein.

Figure S6 Histopathology observation in the gut tissue of kaluga sturgeon

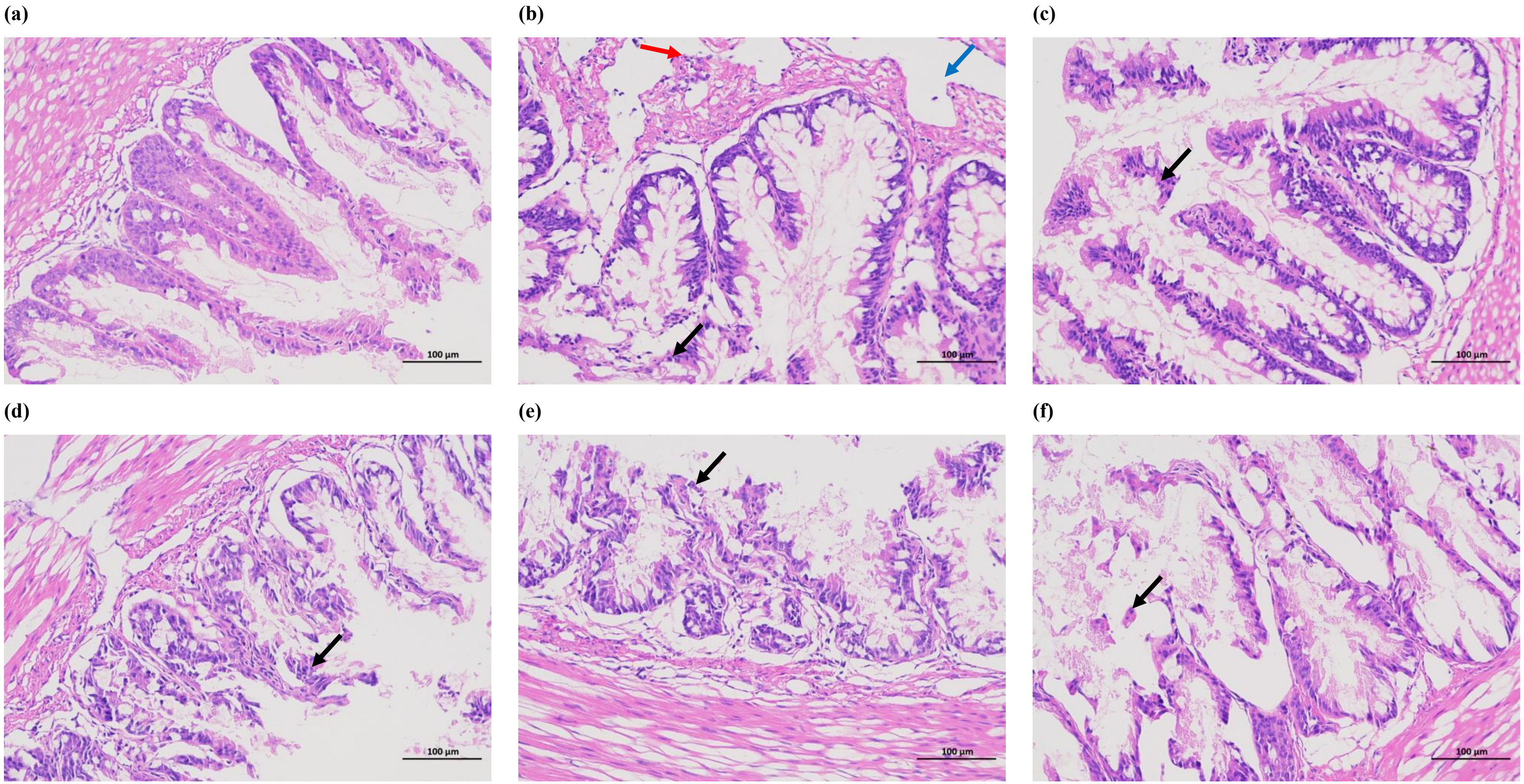

Notes: Each panel showed the intestinal morphology of *Huso dauricus* in (a) 0th d; (b-c) 7th d; (d-e) 14th d and (f) 30th d. Cell nuclei were stained to blue color by hematoxylin while cytoplasm was stained to pink by eosin (200×). Black arrow: shed mucosal epithelial cells. Red arrow: debris residue of necrotic cell. Blue arrow: dilated blood vessel.
